# The Sigma-1 Receptor Is a Novel Target for Improving Cold Preservation in Rodent Kidney Transplants

**DOI:** 10.3390/ijms241411630

**Published:** 2023-07-19

**Authors:** Adam Hosszu, Akos R. Toth, Tamas Lakat, Ganna Stepanova, Zsuzsanna Antal, Laszlo J. Wagner, Attila J. Szabo, Andrea Fekete

**Affiliations:** 1 MTA-SE Lendület “Momentum” Diabetes Research Group, 1083 Budapest, Hungary; hosszu.adam@gmail.com (A.H.);; 2 Pediatric Center, MTA Center of Excellence, Semmelweis University, 1083 Budapest, Hungary; 3 Department of Translational Medicine, Semmelweis University, 1089 Budapest, Hungary; 4 Department of Surgery, Transplantation and Gastroenterology, Semmelweis University, 1082 Budapest, Hungary

**Keywords:** kidney transplantation, organ preservation, renal ischemia/reperfusion injury, Sigma-1 receptor

## Abstract

Kidney transplantation is the preferred treatment for patients with end-stage kidney disease. Maintaining organ viability between donation and transplantation, as well as minimizing ischemic injury, are critically important for long-term graft function and survival. Moreover, the increasing shortage of transplantable organs is a considerable problem; thus, optimizing the condition of grafts is a pivotal task. Here, rodent models of kidney transplantation and cold storage were used to demonstrate that supplementation of a preservation solution with Sigma-1 receptor (S1R) agonist fluvoxamine (FLU) reduces cold and warm ischemic injury. Post-transplant kidney function was improved, histological injury was mitigated, and mRNA expression of two tubular injury markers—kidney injury molecule-1 and neutrophil gelatinase-associated lipocalin—was robustly reduced. In addition, renal inflammation was diminished, as shown by reduced leukocyte infiltration and pro-inflammatory cytokine expression. In the cold ischemia model, FLU ameliorated structural injury profoundly after 2 h as well as 24 h. The reduced number of TUNEL-positive and Caspase 3-positive cells suggests the anti-apoptotic effect of FLU. None of these beneficial effects of FLU were observed in S1R^−/−^ mice. Of note, organ damage in FLU-treated kidneys after 24 h of cold storage was similar to just 2 h without FLU. These results indicate that S1R agonists can prolong storage time and have great potential in improving organ preservation and in alleviating the problem of organ shortages.

## 1. Introduction

Globally, the number of patients receiving treatment for end-stage kidney disease (ESKD) exceeds 750 individuals per million in the general population. The definitive treatment for ESKD is kidney transplantation (KTx), which offers improved survival and quality of life, and reduced medical costs compared to dialysis. ESKD incidence has increased by 35% over the past decade, mainly due to increases in diabetes, hypertension, and the aging of the world population [1]. Consequently, the demand for suitable organs robustly exceeds the available supply [2]. Deceased donor transplants make up two thirds of KTxs, where prolonged periods of cold ischemia are inevitable. Moreover, to keep up with the growing demand for organs, expanded criteria grafts (ECD) are often accepted. Thus, optimizing the condition of expanded-criteria donor grafts and extending graft storage time, which also allows more time for transportation and allocation to obtain better immunological matches, would be pivotal to alleviate donor organ shortage.

Ischemia/reperfusion injury (IRI) remains a major alloantigen-independent obstacle that contributes to primary graft dysfunction, delayed graft function, and graft failure. Transplanted kidneys endure cold ischemia during storage in preservation solutions in the case of deceased donor transplants, as well as warm ischemia during organ harvesting and the transplant surgery. During these ischemic episodes, inflammation, reactive oxygen species production, apoptosis, and necrosis trigger many downstream processes, including vasoconstriction and thrombosis, all of which play a critical role in the pathogenesis of ischemic acute kidney injury (AKI) [3]. Any intervention which prevents or alleviates this injury might decrease the complications caused by IRI. Machine perfusion has advantages compared to static storage, and it is now also well recognized that supplementation of preservation solutions with pharmacologic agents may remarkably improve graft quality.

The Sigma-1 receptor (S1R) is a highly conserved chaperone protein which has mainly been studied in the central nervous system, but is also expressed in peripheral tissues [4,5]. We previously described the S1R as a novel mediator of renoprotective mechanisms in IRI. S1R agonist fluvoxamine (FLU) treatment drastically improved survival and renal function, ameliorated tubular damage, decreased inflammation, and promoted vasodilation after AKI caused by sublethal renal ischemia in rats [6].

Here, in a rat kidney transplant model, we show that perfusion of the donor organ with S1R agonist FLU reduces cold and warm ischemic injury. Thus, organ preservation time can be lengthened, and post-transplant graft condition can be improved.

## 2. Results

### 2.1. S1R Agonist FLU Improves Post-Transplant Kidney Function and Mitigates Tubular Injury

Renal function was substantially impaired 24 h after reperfusion following renal ATx, suggesting the development of ischemia-induced AKI. Serum creatinine and aspartate transaminase (AST) levels in the ATx FLU group indicated milder injury after adding FLU to the preservation solution (Table 1).

**Figure 1:**
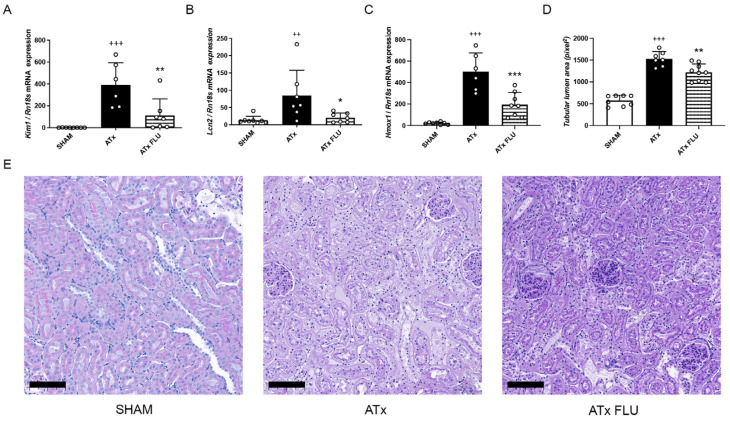
S1R agonist FLU post-transplant kidney injury. mRNA expression levels of (**A**) kidney injury molecule 1 (*Havcr1*), (**B**) neutrophil gelatinase associated lipocalin (*Lcn2*), and (**C**) heme oxygenase 1 (*Hmox1*) genes. (**D**) Mean area of proximal tubular lumen (pixel). (**E**) Representative images of periodic acid-Schiff-stained rat kidney sections (scale bar = 100 µm). ++ *p* < 0.01; +++ *p* < 0.001 vs. SHAM, * *p* < 0.05; ** *p* < 0.01; *** *p* < 0.001 vs. ATx, *n* = 6–8/group.

Histological injury was assessed on PAS-stained kidney sections by measurement of tubular lumen areas. 24 h after ATx tubular damage, cell necrosis and brush border damage were only moderate in kidneys that were stored in a preservation solution containing FLU (ATx FLU) compared to the ATx group, as shown by the diminished tubular lumen area increment (Figure 1D). Moreover, mRNA expression of sensitive and selective tubular injury markers kidney injury molecule-1 (Kim-1; *Havcr1*) and neutrophil gelatinase-associated lipocalin (Ngal; *Lcn2*) were significantly lower when FLU was added to the preservation solution (Figure 1A,B)

Heme oxygenase-1 (HO-1; *Hmox1*) is induced in AKI and has been proposed as a novel biomarker [7]. Congruently, we detected a robust increment of Hmox1 expression after ATx that was mitigated in the ATx FLU group (Figure 1C). These results demonstrate that post-Tx renal function and kidney structure are better preserved when stored in a solution containing an S1R agonist.

### 2.2. FLU Alleviates Inflammation in the Kidney Following ATx

**Figure 2:**
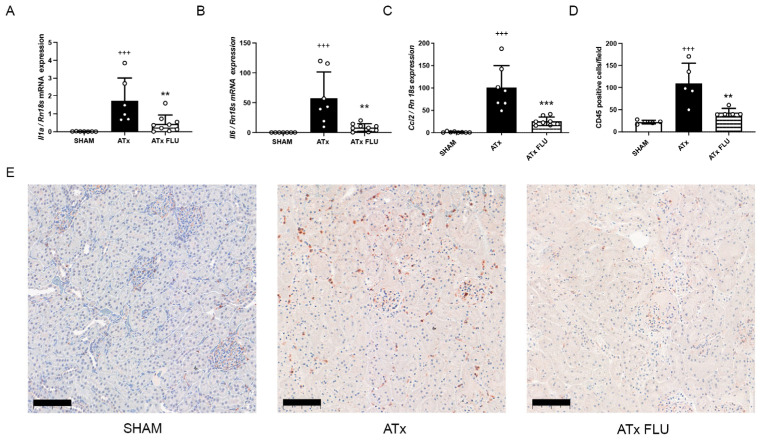
S1R agonist FLU alleviates inflammation in the kidney following ATx. mRNA expression levels of (**A**) interleukin 1 alpha (*Il1a*), (**B**) interleukin 6 (*Il6*), and (**C**) monocyte chemoattractant protein 1 (*Ccl2*). (**D**) Mean number of CD45 positive cells/field of view. (**E**) Representative images of anti-CD45 labeled rat kidney sections (scale bar = 100 µm). +++ *p* < 0.001 vs. SHAM, ** *p* < 0.01; *** *p* < 0.001 vs. ATx, *n* = 6–8/group.

Anti-CD45 immunohistochemical staining revealed a massive reduction in leukocyte infiltration in kidneys stored in a preservation solution containing FLU. In parallel, monocyte chemoattractant protein 1 (Mcp-1; *Ccl2*), Il-1α, and Il-6 mRNA expressions were diminished in these kidneys following ATx compared to ones stored in a preservation solution without FLU (Figure 2).

### 2.3. Kidneys Are Better Preserved during Cold Storage with the Addition of FLU to the Preservation Solution

**Figure 3:**
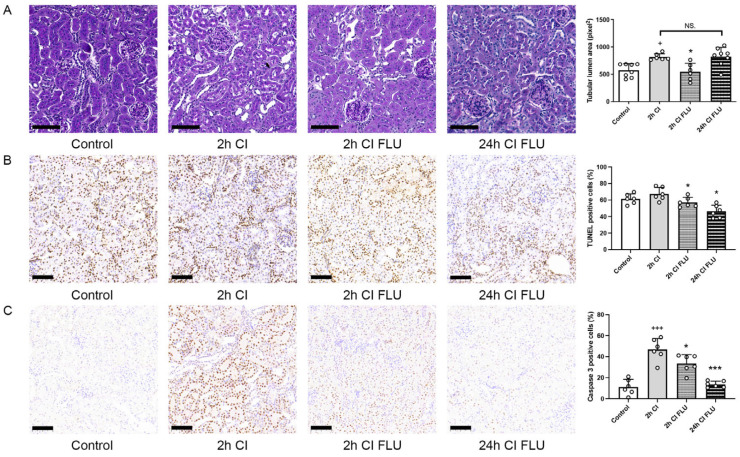
Kidneys are better preserved during cold storage with the addition of FLU to the preservation solution. (**A**) Mean area of proximal tubular lumen (pixel) evaluated on periodic acid-Schiff-stained rat kidney sections. (**B**) Mean number of apoptotic cells evaluated on rat kidney sections labeled with TUNEL assay. (**C**) Mean number of Caspase-3 positive cells/field of view evaluated on anti-Caspase-3 labeled rat kidney sections (scale bar = 100 µm). + *p* < 0.05; +++ *p* < 0.001 vs. Control, * *p* < 0.05; *** *p* < 0.001 vs. 2 h CI, NS. = not significant, *n* = 6–8/group.

Kidneys were perfused and stored in ice cold preservation solutions for various time periods to mimic cold storage before a deceased donor transplantation. As expected, structural injury was apparent after 2 h of cold storage (2 h CI). The addition of FLU to the preservation solution mitigated the progression of structural injury so profoundly, that cold storage-induced organ damage after just 2 h without FLU was similar to 24 h with FLU (24 h CI FLU) (Figure 3A). The reduced number of TUNEL-positive and Caspase 3-positive cells after both 2 and 24 h of cold storage shows the anti-apoptotic effect of FLU (Figure 3B,C). These results indicate that with the addition of FLU, storage time can be significantly lengthened without additional renal damage.

### 2.4. The Beneficial Effects of FLU Are Diminished in S1R^−/−^ Mice

**Figure 4:**
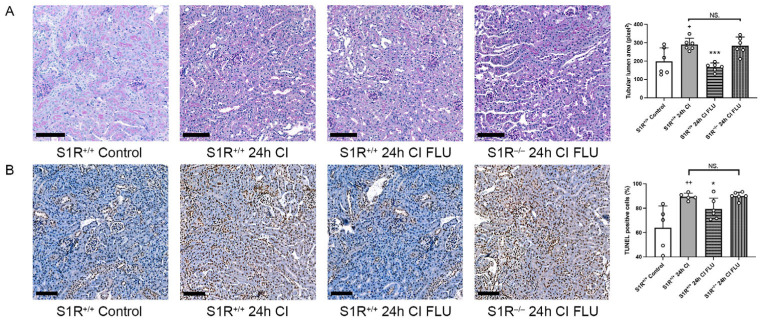
Kidneys are better preserved during cold storage with the addition of FLU to the preservation solution in S1R^+/+^, but not in S1R^−/−^ mice. (**A**) Mean area of proximal tubular lumen (pixel) evaluated on periodic acid-Schiff-stained mouse kidney sections. (**B**) Mean number of apoptotic cells evaluated on mouse kidney sections labeled with TUNEL assay (scale bar = 100 µm). + *p* < 0.05; ++ *p* < 0.01 vs. Control, * *p* < 0.05; *** *p* < 0.001 vs. 2 h CI, NS. = not significant, *n* = 5–6/group.

To confirm that renoprotective mechanisms are mediated by S1R per se, the cold ischemia model was repeated on S1R^+/+^ and S1R^−/−^ mice. Tubular structural injury, as well as the number of apoptotic cells after 24 h of cold ischemia, was alleviated by FLU in S1R^+/+^, but not in in S1R^−/−^ mice, which supports our hypothesis (Figure 4).

## 3. Discussion

IRI during KTx is a major alloantigen-independent factor that determines graft and patient survival, and each hour of cold ischemia significantly increases the risk of DGF [8]. In the present study, we show that pretransplant perfusion and storage of the donor organ with a S1R agonist improves transplant outcomes by reducing functional decrement, tubular injury, renal inflammation, and apoptosis. Together, these findings unveil the S1R as a novel therapeutic target in cold storage KTx that may also alleviate donor shortage by improving the condition of ECD organs by extending cold storage times.

S1R is a highly conserved chaperone protein which has mainly been studied in the central nervous system but is also expressed in peripheral tissues. It is a multifunctional inter-organelle signaling molecule that plays diverse roles in cellular survival [4,5]. We recently described the S1R as a novel mediator of renoprotective mechanisms in IRI. We were the first to specify the exact renal localization of the receptor and to outline a new molecular mechanism by which up-regulated Akt-eNOS signaling results in peritubular capillary dilatation and thus improved post-ischemic renal perfusion [6]. Moreover, we showed that in females, S1R activation by estrogen enhances the heat shock response in the kidney after an ischemic event, and thus contributes to the relative renoprotection females enjoy compared to males [9].

Based on our recent studies, a rat model of kidney autotransplantation with cold ischemia was employed here, which allows for the investigation of ischemic injury without the risk of rejection or the possible confounding side effects of immunosuppressive drugs. Moreover, we aimed to mimic deceased donor transplants, which are inherently associated with cold IRI and account for more than 70% of all KTx’s [10].

Post-ATx histological injury as well as renal function was improved in rats with kidneys perfused with FLU, as shown by decreased serum creatinine and tubular injury marker AST levels [11]. Recent papers widely discuss the limited diagnostic accuracy of traditional markers of AKI, and the superior sensitivity and specificity of biomarkers such as Kim-1 and Ngal. Warm and cold ischemia result in different patterns of injury: warm ischemia drives a proximal tubular pattern, whereas cold ischemia causes distal nephron injury [12]. Kim-1 is expressed by proximal tubular cells, and strongly correlates with the severity of histological injury [13], while Ngal is primarily expressed in the distal nephron [14]. Here, as expected, the expression of both genes was robustly increased after ATx, which was decreased by 75% in FLU-treated ATx kidneys.

HO-1 is a stress-responsive enzyme, known to be up-regulated in the kidney by various stimuli including hypoxia, heat shock, and toxins [15]. Accumulating evidence indicates that HO-1 overexpression provides cytoprotective effects and improves kidney function following AKI [16]. HO-1-deficient mice express significantly higher levels of pro-inflammatory IL-6 while producing low levels of the anti-inflammatory IL-10 [17]. In our results, the lower expression of HO-1 mRNA in FLU-treated kidneys indicates milder injury; however, HO-1 is likely not involved in S1R-mediated renoprotection.

Low-grade, systemic inflammation has been shown to be a risk factor for overall mortality after KTx [18]. Moreover, early inflammation and leucocyte recruitment are critical mediators of tubular injury following IRI. Injured proximal tubular cells, leucocytes, as well as macrophages produce pro-inflammatory cytokines and chemokines which exacerbates injury [19]. The anti-inflammatory effect of S1R activation has been extensively demonstrated by us and others in both cell and animal models [6,20,21]; thus, as expected, FLU attenuated CD45+ leucocyte infiltration and pro-inflammatory cytokine IL-1α, IL-6, and MCP-1 expressions following ATx.

To meet the constantly growing organ demands, grafts retrieved from ECDs or donated from DCD (donation-after-death, non-heart-beating) donors or are increasingly utilized. Unfortunately, urine production and filtration capacity are often reduced in these grafts, which increases post-transplant complications and mortality [22].

Kayler et al. analyzed more than 18 thousand KTx recipients and concluded that even a ≥1-h increase in cold ischemia time predisposes to delayed graft function, which means that truly every minute counts [23]. Static cold storage remains the gold standard for renal graft preservation based on its availability and superiority in lowering metabolism and preserving viability, and was therefore used in the present study. Conversely, hypothermia causes reduced ATP production and reduced Na^+^/K^+^-ATPase activity, which results in mitochondrial and osmotic disruption, and in turn induces renal cell death. Indeed, even 2 h of CI provoked significant histological injury and apoptosis in our experimental model. Importantly, with the addition of S1R agonist FLU to the preservation solution, injury after 24 h of CI was similar to after just 2 h without FLU; thus, storage time can be lengthened without additional injury, and the condition of grafts can be optimized. This effect was not observed in S1R^−/−^ mice, which confirms that the renoprotection exerted by FLU is directly S1R-mediated.

In recent years, some progress has been made in the field of organ preservation. Hypothermic machine perfusion has been shown to be associated with a significantly lower DGF rate, but this positive effect wanes with cold ischemia times longer than 10 h [24]. The impact of cold ischemia time could be targeted by normothermic machine perfusion, which has shown some promising results in several studies [25,26]. Although dynamic preservation was not performed in our study, one can assume that S1R agonists exert their renoprotective effects in any organ preservation technique and preservation solution.

In summary, novel strategies are desperately needed to alleviate kidney injury in cold-storage-associated transplantation. Our results suggest that supplementation of the preservation solution with FLU ameliorates functional decrement, tubular injury, renal inflammation, and apoptosis. Thus, S1R agonists have great potential in organ preservation and the alleviation of organ shortages.

## 4. Materials and Methods

Animal procedures were approved by the Committee on the Care and Use of Laboratory Animals of the Semmelweis University Budapest, Hungary (PEI/001/1731-9-2015).

### 4.1. Materials

All chemicals and reagents were purchased from Sigma-Aldrich (St. Luis, MO, USA), and all standard plastic laboratory equipment was purchased from Sarstedt (Numbrecht, Germany) unless stated otherwise.

### 4.2. Animals

Experiments were performed on male Han Wistar rats weighing 250 ± 25 g (Toxi-Coop Ltd., Budapest, Hungary), or wild-type (S1R^+/+^) and S1R-knockout (S1R^−/−^) mice weighing 25 ± 2 g. Mice were of a C57BL/6j × 129 s/Sv mixed background and kindly gifted from Dr. Adrian Wong (Ottawa Hospital Research Institute, Ottawa, ON, Canada). The mice used in the experiments were produced from heterozygous breeding pairs and assigned randomly to each experiment. The S1R^−/−^ and S1R^+/+^ genotypes were confirmed by PCR. Animals were housed in groups of three or six under a 12-h light-dark cycle at 22 ± 2 °C with free access to standard rodent chow and tap water.

### 4.3. Experimental Protocols

#### 4.3.1. Renal Autotransplantation (ATx)

Rats were randomly assigned to one of the following groups: (1) SHAM, *n* = 8, sham operated controls; (2) ATx, *n* = 6–8, uninephrectomized, received kidney perfused with ice-cold Custodiol (Dr. Franz Kohler Chemie Gmbh., Bensheim, Germany) preservation solution; (3) ATx FLU, *n* = 6–8, uninephrectomized, received kidney perfused with 4 °C Custodiol preservation solution containing 10 µM fluvoxamine maleate (FLU, Sigma Aldrich, Budapest, Hungary).

General anesthesia was induced by intraperitoneal administration of ketamine (75 mg × bwkg^−1^, Richter Gedeon Plc., Budapest, Hungary) and xylasine (10 mg × bwkg^−1^, Medicus Partner, Biatorbagy, Hungary). Renal autotransplantation was performed as previously described [27]. Briefly, the kidneys and vessels were approached through a midline incision, and atraumatic vascular clips were placed on the left renal artery and vein. The left kidney was perfused with 4 °C Custodiol or Custodiol containing FLU, and then stored at 4 °C in the same solution for 2 h before being transplanted back into the same animal (autotransplantation: ATx). After exactly 35 min of warm ischemia, the contralateral kidney was removed, the abdomen was closed in layers, and animals were placed back in their cages. Rats were re-anaesthetized, blood samples were collected from the abdominal aorta, and kidney samples were collected 24 h after reperfusion.

#### 4.3.2. Cold Ischemia (CI)

Rats were randomly assigned to one of the following groups: (1) Custodiol 2 h of cold ischemia (2 h CI); (2) Custodiol + 10 µM FLU 2 h of CI (2 h CI FLU); (3) Custodiol + 10 µM FLU 24 h of CI (24 h CI FLU).

Mice were randomly assigned to one of the following groups: (1) wild-type Custodiol 24 h of CI (S1R^+/+^ 24 h CI); (2) wild-type Custodiol + 10 µM FLU 24 h of CI (S1R^+/+^ 24 h CI FLU); (3) S1R knockout Custodiol + 10 µM FLU 24 h of CI (S1R^−/−^ 24 h CI FLU).

The renal artery was cannulated, and the kidneys were first flushed with 4 °C isotonic saline for 1 min. Next, the kidneys were perfused with 200 mL 4 °C Custodiol or Custodiol + 10 µM FLU with a perfusion pressure of 80–100 mmHg and placed on ice in the same solution for 2 or 24 h before being collected.

### 4.4. Histology

Kidneys were fixed in 4% buffered formaldehyde, embedded in paraffin, and 5 µm thick sections were cut. Renal structural injury (specifically dilation of proximal tubules with loss or thinning of brush border) was evaluated semi-quantitatively on periodic acid-Schiff (PAS)-stained sections and quantified as follows: five square areas of 200× magnification were selected on each kidney section with Panoramic Viewer software (software version 1.15.4., 3DHISTECH, Budapest, Hungary) [28]. Tubular lumens were colored black, and the numbers of pixels were measured and averaged per kidney using Adobe Photoshop CS6 software (Adobe Inc., San Jose, CA, USA). The evaluation was performed in a blinded fashion by a transplant pathologist.

### 4.5. Apoptosis Detection by TUNEL Assay

5 µm thick sections were mounted on Superfrost slides (Thermo Shandon, Runcorn, UK) and were manually deparaffinized. Assay was performed using the Apoptag^®^ Peroxidase in situ Apoptosis Detection Kit (Millipore, Billerica, MA, USA). Briefly, samples were pretreated with Proteinase K for 15 min. Following repeated washing steps, endogenous peroxidase activity was blocked by 3% H_2_O_2_ in methanol for 5 min at room temperature (RT). Next, slides were incubated in a reaction buffer containing 30% TdT enzyme for 1 h at RT, after which a stop buffer was added. Slides were incubated with anti-Dioxigenin Conjugate for 30 min at RT. Slides were developed using a DAB peroxidase substrate. TUNEL-positive apoptotic nuclei were counted in five fields of magnification ×200 per kidney. The analysis was performed using Adobe Photoshop CS6 (Adobe Inc., San Jose, CA, USA) and Image J (software version 1.54a, National Institute of Health, Bethesda, MD, USA) software.

### 4.6. CD45 Assay

1 μm thick sections were deparaffinized manually. Endogenous peroxidase activity was blocked with a 3% hydrogen peroxidase–methanol mixture for 20 min at RT. Slides were rinsed in 0.05 mM citrate buffer (pH = 6.0) and incubated at 93 °C for 10 min. The sections were then incubated overnight at 4 °C with the primary anti-CD45 antibody (Abcam ab10558, Cambridge, UK) diluted (1:100) in PBS. After washes, slides were incubated with the biotinylated secondary antibody (DakoCytomation, Glostrup, Denmark) for 15 min at RT. Visualization was performed with the avidin-biotin peroxidase complex method (ABC system, Dako) using aminoethyl carbazole as the chromogen. The extent of interstitial leukocyte infiltration was evaluated quantitatively on total kidney sections using Qupath software version 0.1.2 (Centre for Cancer Research & Cell Biology, Queen’s University, Belfast, UK).

### 4.7. RT-qPCR

Total RNA was extracted with Total RNA isolation Mini Kit (Geneaid Biotech, New Taipei City, Taiwan) according to the manufacturer’s instructions. The quality and quantity of isolated RNA were measured on the NanoDrop ND-1000 spectrophotometer (BCM, Huston, TX, USA). First strand cDNA was prepared from total RNA using the Maxima First Strand cDNA Synthesis Kit for RT-qPCR (Thermo Fisher Scientific, Waltham, MA, USA). Real-time RT-qPCR was performed on a LightCycler 96 system (Roche Diagnostics, Mannheim, Germany) using 1 μL cDNA samples, 10 μL SYBR Green I Master enzyme mix (Roche Diagnostics, Mannheim, Germany), and 10 pmol μL^−1^ of each specific primer (IDT, Coralville, IA, USA). Results were analyzed by the LightCycler 96 software version 1.1.0.1320 (Roche Diagnostics, Mannheim, Germany). All data were normalized to the expression of Rn18s housekeeping gene from the same samples as the reference transcript. Primer sequences are listed in Table 2.

## 5. Patents

Andrea Fekete, Adam Vannay, Adam Hosszu. Compositions for organ preservation: European Patent Office EP3544420A1, filed 24 November 2017, issued 2 October 2019. The invention relates to use of a Sigma 1 receptor agonist compound in preservation solutions and preservation solutions comprising a Sigma 1 receptor agonist compound to preserve the viability of organs.

## Figures and Tables

**Table 1:** Renal functional parameters are improved by fluvoxamine (FLU) following autotransplantation (ATx).

	SHAM	ATx	ATx FLU
Creatinine (µmol/L)	28.3 ± 6.2	361.7 ± 42 ^+++^	308.2 ± 42.6 ^+++,^*
BUN (mmol/L)	7.5 ± 1.8	55.5 ± 6.5 ^+++^	57 ± 7.7 ^+++^
AST (U/L)	233.8 ± 40	507.8 ± 180.8 ^+++^	294.3 ± 118.2 ***
Na (mmol/L)	142.8 ± 2.5	137.9 ± 3.1	137.2 ± 2.7
K (mmol/L)	6.9 ± 1	7.9 ± 0.8	8.2 ± 0.5
Cl (mmol/L)	103.2 ± 2.5	95.2 ± 4.1	93.2 ± 4.7

+++ *p* < 0.001 vs. SHAM, * *p* < 0.05; *** *p* < 0.001 vs. ATx, *n* = 6–8/group. BUN: blood urea nitrogen; AST: aspartate transaminase.

**Table 2:** RT-qPCR primer sequences used in this study.

Gene Name	Regular Name	NCBI ID	Primer Sequence	Product Length (bp)
*Rn18s*	Rat 18S ribosomal RNA	100861533	F: 5′ GCGGTCGGCGTCCCCCAACTTCTT 3′	105
			R: 5′ GCGCGTGCAGCCCCGGACATCTA 3′	
*Il1a*	Rat IL-1α	24493	F: 5′ GGCTGAGAAAGAGGAGTTCG 3′	152
			R: 5′ CCACCCATCTGTCTCCTAGA 3′	
*Il6*	Rat IL-6	24498	F: 5′ GCCACTGCCTTCCCTACTTC 3′	153
			R: 5′ GCCATTGCACAACTCTTTTCTC 3′	
*Havcr1*	Rat KIM-1	286934	F: 5′ CGCAGAGAAACCCGACTAAG 3′	194
			R: 5′ CAAAGCTCAGAGAGCCCATC 3′	
*Lcn2*	Rat NGAL	170496	F: 5′ CAAGTGGCCGACACTGACTA 3′	194
			R: 5′ GGTGGGAACAGAGAAAACGA 3′	
*Hmox1*	Rat HO-1	24451	F: 5′ AGACCGCCTTCCTGCTCAACATT 3′	160
			R: 5′ CATTTTCCTCGGGGCGTCTCTG 3′	
*Ccl2*	Rat MCP-1	24770	F: 5′ ATGCAGTTAATGCCCCACTC 3′	167
			R: 5′ TTCCTTATTGGGGTCAGCAC 3′	

## Data Availability

Data is contained within the article.

## References

[1] Thurlow J.S., Joshi M., Yan G., Norris K.C., Agodoa L.Y., Yuan C.M., Nee R. (2021). Global Epidemiology of End-Stage Kidney Disease and Disparities in Kidney Replacement Therapy. Am. J. Nephrol..

[2] Tullius S.G., Rabb H. (2018). Improving the Supply and Quality of Deceased-Donor Organs for Transplantation. N. Engl. J. Med..

[3] Requião-Moura L.R., Durão Mde S., Tonato E.J., Matos A.C., Ozaki K.S., Câmara N.O., Pacheco-Silva A. (2011). Effects of ischemia and reperfusion injury on long-term graft function. Transplant. Proc..

[4] Hellewell S.B., Bruce A., Feinstein G., Orringer J., Williams W., Bowen W.D. (1994). Rat liver and kidney contain high densities of sigma 1 and sigma 2 receptors: Characterization by ligand binding and photoaffinity labeling. Eur. J. Pharmacol..

[5] Ela C., Barg J., Vogel Z., Hasin Y., Eilam Y. (1994). Sigma receptor ligands modulate contractility, Ca++ influx and beating rate in cultured cardiac myocytes. J. Pharmacol. Exp. Ther..

[6] Hosszu A., Antal Z., Lenart L., Hodrea J., Koszegi S., Balogh D.B., Banki N.F., Wagner L., Denes A., Hamar P. (2017). sigma1-Receptor Agonism Protects against Renal Ischemia-Reperfusion Injury. J. Am. Soc. Nephrol..

[7] Zager R.A., Johnson A.C., Becker K. (2012). Plasma and urinary heme oxygenase-1 in AKI. J. Am. Soc. Nephrol. JASN.

[8] Debout A., Foucher Y., Trébern-Launay K., Legendre C., Kreis H., Mourad G., Garrigue V., Morelon E., Buron F., Rostaing L. (2015). Each additional hour of cold ischemia time significantly increases the risk of graft failure and mortality following renal transplantation. Kidney Int..

[9] Hosszu A., Antal Z., Veres-Szekely A., Lenart L., Balogh D.B., Szkibinszkij E., Illesy L., Hodrea J., Banki N.F., Wagner L. (2018). The role of Sigma-1 receptor in sex-specific heat shock response in an experimental rat model of renal ischaemia/reperfusion injury. Transpl. Int..

[10] Lentine K.L., Smith J.M., Hart A., Miller J., Skeans M.A., Larkin L., Robinson A., Gauntt K., Israni A.K., Hirose R. (2022). OPTN/SRTR 2020 Annual Data Report: Kidney. Am. J. Transplant. Off. J. Am. Soc. Transplant. Am. Soc. Transpl. Surg..

[11] Chatterjee P.K., Cuzzocrea S., Brown P.A., Zacharowski K., Stewart K.N., Mota-Filipe H., Thiemermann C. (2000). Tempol, a membrane-permeable radical scavenger, reduces oxidant stress-mediated renal dysfunction and injury in the rat. Kidney Int..

[12] Desanti De Oliveira B., Xu K., Shen T.H., Callahan M., Kiryluk K., D’Agati V.D., Tatonetti N.P., Barasch J., Devarajan P. (2019). Molecular nephrology: Types of acute tubular injury. Nat. Rev. Nephrol..

[13] Vaidya V.S., Ozer J.S., Dieterle F., Collings F.B., Ramirez V., Troth S., Muniappa N., Thudium D., Gerhold D., Holder D.J. (2010). Kidney injury molecule-1 outperforms traditional biomarkers of kidney injury in preclinical biomarker qualification studies. Nat. Biotechnol..

[14] Liu J., Krautzberger A.M., Sui S.H., Hofmann O.M., Chen Y., Baetscher M., Grgic I., Kumar S., Humphreys B.D., Hide W.A. (2014). Cell-specific translational profiling in acute kidney injury. J. Clin. Investig..

[15] Bolisetty S., Zarjou A., Agarwal A. (2017). Heme Oxygenase 1 as a Therapeutic Target in Acute Kidney Injury. Am. J. Kidney Dis..

[16] Ferenbach D.A., Kluth D.C., Hughes J. (2010). Hemeoxygenase-1 and renal ischaemia-reperfusion injury. Nephron Exp. Nephrol..

[17] Tracz M.J., Juncos J.P., Croatt A.J., Ackerman A.W., Grande J.P., Knutson K.L., Kane G.C., Terzic A., Griffin M.D., Nath K.A. (2007). Deficiency of heme oxygenase-1 impairs renal hemodynamics and exaggerates systemic inflammatory responses to renal ischemia. Kidney Int..

[18] Abedini S., Holme I., März W., Weihrauch G., Fellström B., Jardine A., Cole E., Maes B., Neumayer H.H., Grønhagen-Riska C. (2009). Inflammation in renal transplantation. Clin. J. Am. Soc. Nephrol..

[19] Sharfuddin A.A., Molitoris B.A. (2011). Pathophysiology of ischemic acute kidney injury. Nat. Rev. Nephrol..

[20] Rosen D.A., Seki S.M., Fernández-Castañeda A., Beiter R.M., Eccles J.D., Woodfolk J.A., Gaultier A. (2019). Modulation of the sigma-1 receptor-IRE1 pathway is beneficial in preclinical models of inflammation and sepsis. Sci. Transl. Med..

[21] Rafiee L., Hajhashemi V., Javanmard S.H. (2016). Fluvoxamine inhibits some inflammatory genes expression in LPS/stimulated human endothelial cells, U937 macrophages, and carrageenan-induced paw edema in rat. Iran. J. Basic Med. Sci..

[22] Abou Taka M., Dugbartey G.J., Sener A. (2022). The Optimization of Renal Graft Preservation Temperature to Mitigate Cold Ischemia-Reperfusion Injury in Kidney Transplantation. Int. J. Mol. Sci..

[23] Kayler L.K., Srinivas T.R., Schold J.D. (2011). Influence of CIT-induced DGF on kidney transplant outcomes. Am. J. Transplant. Off. J. Am. Soc. Transplant. Am. Soc. Transpl. Surg..

[24] Kox J., Moers C., Monbaliu D., Strelniece A., Treckmann J., Jochmans I., Leuvenink H., Van Heurn E., Pirenne J., Paul A. (2018). The Benefits of Hypothermic Machine Preservation and Short Cold Ischemia Times in Deceased Donor Kidneys. Transplantation.

[25] Kaths J.M., Echeverri J., Goldaracena N., Louis K.S., Chun Y.M., Linares I., Wiebe A., Foltys D.B., Yip P.M., John R. (2016). Eight-Hour Continuous Normothermic Ex Vivo Kidney Perfusion Is a Safe Preservation Technique for Kidney Transplantation: A New Opportunity for the Storage, Assessment, and Repair of Kidney Grafts. Transplantation.

[26] Weissenbacher A., Vrakas G., Nasralla D., Ceresa C.D.L. (2019). The future of organ perfusion and re-conditioning. Transpl. Int. Off. J. Eur. Soc. Organ Transplant..

[27] Tillou X., Howden B.O., Kanellis J., Nikolic-Paterson D.J., Ma F.Y. (2016). Methods in renal research: Kidney transplantation in the rat. Nephrology.

[28] Hanif M.O., Bali A., Ramphul K. (2023). Acute Renal Tubular Necrosis. StatPearls.

